# A Treatise on Surgery

**Published:** 1906-04

**Authors:** 


### BOOK REVIEWS.

A TREATISE ON SURGERY. In two volumes. By George R. Fowler, M.D., Examiner in Surgery, Board of Medical Examiners of the Regents of the University of the State of New York; Emeritus Professor of Surgery in the New York Polyclinic, etc. Two imperial octavos of 725 pages each, with 888 text illustrations and four colored plates, all original. Philadelphia and London: W. B. Saunders Company, 1906. Per set: Cloth, $15.00 net; half morocco, $17.00 net.

We have been looking forward to the appearance of this work with the greatest expectations, for Dr. Fowler's endeavors in the field of practical surgery have been such as to stamp his writings with unquestionable authority. It is not too much, indeed, we feel it is too little to say that our expectations have been fully realized. The work is a masterpiece. It is an accurate, up-to-date treatise on surgery, skilfully presented. This entirely new work presents the science and art of surgery as it is practiced to-day. The first part of the work deals with general surgery, and embraces what is usually included under the head of principles of surgery. Special attention is given to the subject of inflammation from the surgeon's point of view, due consideration being accorded the influences of traumatism and bacterial infection as the predisposing and exciting causes of this condition. Then follow sections on the injuries and diseases of separate tissues, gunshot injuries, acute wound diseases, chronic surgical infections (including syphilis), tumors, surgical operations in general, foreign bodies, and bandaging. The second part of the work is really the clinical portion, devoted to regional surgery. Herein the author especially endeavors to emphasize those injuries and surgical diseases that are of the greatest importance, not only because of their frequency, but also because of the difficulty of diagnosis and the special care demanded in their treatment. Throughout special attention has been given to diagnosis, the section on laboratory aids being unusually excellent. The text is elaborately illustrated with entirely new and original illustrations, and evidently neither labor nor expense has been spared to bring this feature of the work up to the highest standard of artistic and practical excellence.

